# Fabrication of Microneedles by Pulsating In Situ Dried Electrostretching for Transdermal Drug Delivery

**DOI:** 10.1002/smtd.202500183

**Published:** 2025-05-07

**Authors:** Ngoc Luan Mai, Yuen Yong, Thi Van Anh Hoang, Trung Hieu Vu, Hoai‐Duc Vu, Van Canh Doan, Donglin Cai, Thien Xuan Dinh, Dzung Viet Dao, Van Thanh Dau

**Affiliations:** ^1^ School of Engineering and Built Environment Griffith University Queensland 4215 Australia; ^2^ School of Engineering The University of Newcastle NSW 2308 Australia; ^3^ School of Medicine and Dentistry Griffith University Queensland 4215 Australia; ^4^ The Commonwealth Scientific and Industrial Research Organisation Clayton 3168 Australia

**Keywords:** electrostretching, polymeric microneedles, pulsed voltage, solvent evaporation, transdermal drug delivery

## Abstract

This paper introduces a novel pulsating in situ dried electrostretching (PIDES) technique for the fabrication of microneedles (MNs) for transdermal drug delivery. This method utilizes pulsed voltage to induce electrohydrodynamic forces that stretch and freeze a polymer droplet into a conical shape with a micrometer‐scale tip. With the effects of solvent evaporation, the polymeric droplet is in situ stretched into a conical shape and solidified, transforming into a sharp MN, suitable for transdermal drug administration. Penetration and mechanical tests confirm that the MNs possess sufficient sharpness and strength for effective skin penetration applications. Additionally, curcumin loading and in vitro release tests with different concentrations demonstrate the MNs' ability to carry drugs and exhibit effective controlled release profiles. These findings highlight PIDES as a promising, low‐cost, and simple approach for the development of painless and efficient transdermal drug delivery systems.

## Graphical abstract

The pulsating in situ dried electrostretching (PIDES) technique offers a novel approach to microneedles fabrication for transdermal drug delivery utilizing electrohydrodynamic principles. PIDES is capable of fabricating biodegradable polymeric microneedles with geometric and mechanical properties suitable for skin penetration. Drug loading and in vitro release tests confirm a controlled release profile for different curcumin concentrations, thus proving the pronounced potential of PIDES.

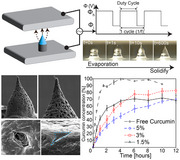

## 1 Introduction

Microneedle (MN) is a cutting‐edge technology in the field of transdermal drug delivery. It offers a painless, convenient alternative method to subcutaneous injection by conventional hypodermic needles. MNs are designed to be less than 1 mm in length with a sharp tip to penetrate the outermost skin layer (stratum corneum) without reaching and stimulating the nerve endings.^[^ ^1^, ^2^ ^]^ MNs have been proven useful in a wide range of medical applications, from transdermal administration of drugs,^[^ ^3^, ^4^, ^5^, ^6^, ^7^ ^]^ vaccines,^[^ ^8^, ^9^ ^]^ therapeutic enzymes,^[^ ^10^ ^]^ beneficial bacteria,^[^ ^11^ ^]^ to hair and wound regeneration,^[^ ^12^, ^13^, ^14^, ^15^ ^]^ blood extraction^[^ ^16^ ^]^ and human‐machine interfaces (HMIs).^[^ ^17^, ^18^, ^19^, ^20^ ^]^

Micro‐molding is considered the most common method to fabricate microneedles.^[^ ^21^ ^]^ This technique comprises several steps, beginning with the master molds, compressing the MNs materials into the molds by vacuum, centrifuge, heated imprinting or spin coating,^[^ ^6^, ^15^, ^17^, ^22^, ^23^, ^24^ ^]^ followed by curing and demolding the solidified MNs. These processes may require high temperature,^[^ ^9^ ^]^ which poses challenges for drug encapsulation due to temperature sensitivity. Micro‐molding also enables the fabrication of multi‐layered MNS by multi‐step filling of materials into the negative mold.^[^ ^6^, ^25^ ^]^ This technique helps facilitate different drug release schematics as well as improve attachability.^[^ ^26^ ^]^ Recently, wet etching has arisen as a successful fabrication technique for MNs, which involves isotropically etching a Si wafer into MN array. The obtained array can be used as a base structure to deposit conductive materials for physiological electrodes,^[^ ^18^, ^27^ ^]^ or to cast the micromold.^[^ ^28^ ^]^ Although mold‐based methods possess high accuracy, reusability, and allow for fabricating multicomponent microneedles or mass production. They are constrained by inefficient multi‐stage processes, inflexible molds, and drug loss. Moldless methods have arisen to address these issues. Liquid drawing technique, utilizing the elastocapillary self‐thinning of the extensional elongation of liquid^[^ ^29^ ^]^ or thermoplastic behavior of metallic glass^[^ ^30^ ^]^ to fabricate MNs, has been reported. This method can be assisted by an external magnetic field to maintain the MN shape^[^ ^31^ ^]^ or additional airflow to enhance solvent evaporation.^[^ ^32^ ^]^ In addition, fabrication of conical and pyramidal MN was successfully demonstrated via 3D printing stereolithography for transdermal delivery of insulin.^[^ ^33^ ^]^ Another noteworthy technique is the utilization of the pyro‐electrohydrodynamic force induced by a thermally stimulated polar dielectric crystal to fabricate MNs from sessile drops of biopolymer solution.^[^ ^4^, ^7^ ^]^ However, these methods still require improvements as they can be limited by low accuracy, time‐consuming processes and the requirements for precision instrumentation.

In this paper, we propose a novel microneedles fabrication technique by using pulsating in situ dried electrostretching (PIDES), in which the polymeric droplet is deformed and stretched into MNs by pulsed electrohydrodynamic (EHD) force, dried and solidified by diffusive solvent evaporation. This technique offers a simple and contactless fabrication process which would minimize drug loss and drug degradation. To demonstrate the proposed technique, we designed a parallel electrode system and fabricated MNs from a polycaprolactone (PCL) solution. Results showed that the achieved MNs are suitable for transdermal drug delivery in terms of sharpness, overall dimensional features, and process repeatability. Penetration test in agarose gel and compressive mechanical tests further validated the strength of the electrostretched MNs. Curcumin loading and in vitro release results demonstrated the effective loading capability and controlled release profile of drug of our method. We also developed a numerical model to explain the physical principles underlying our technique and provide parametrical correlation predictions for future studies.

## 2 PIDES Concept

**Figure 1:**
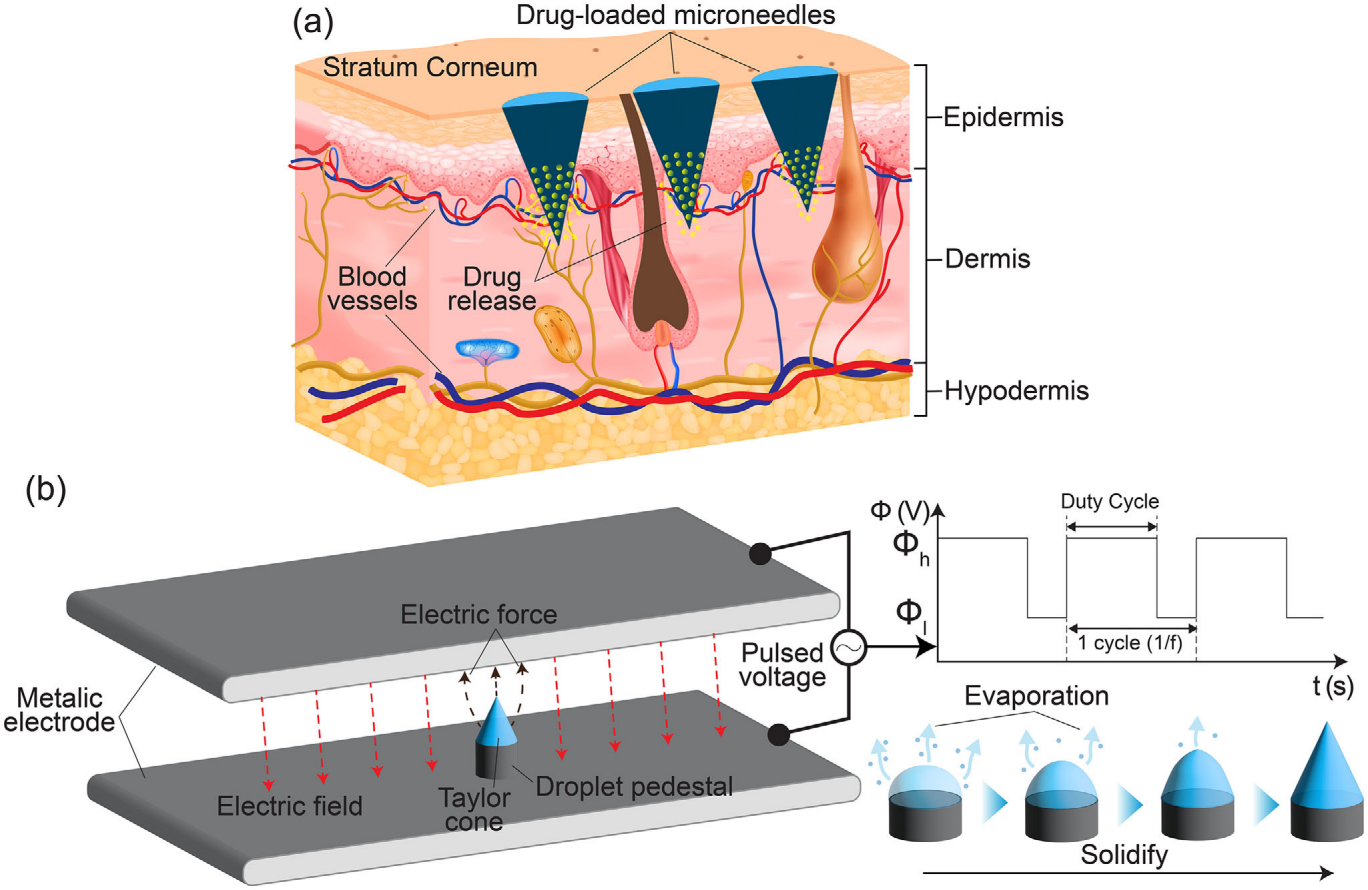
a) Illustration of transdermal drug delivery by drug‐loaded MNs, the MNs are required to penetrate the dermis layer, releasing drug particles into the blood vessels without causing pain; b) The working principle of the parallel‐plate electrode apparatus alongside with the illustrations of the pulsed voltage behavior and the deformation‐solidification process of the polymeric droplet.

**Figure** **1**a shows the working principle of the MNs in a cross‐sectional schematic of the human skin. The ideal MNs should be able to penetrate through the stratum corneum and the epidermis layer of the skin, which are around 10–20 and 60–800 µm in thickness, respectively.^[^ ^34^ ^]^ Drug can be either encapsulated inside or coated on the surface of the MNs before controllably releasing into the body.^[^ ^21^ ^]^ Figure 1b. illustrates our PIDES concept using two parallel electrodes connected to an AC high‐voltage power source. A polymeric solution is placed within the interelectrode. Under the strong electric field, the droplet is polarized and electrically charged, which exerts an interfacial force forming a conical shape commonly known as the Taylor cone. The upper electrode is connected to a high voltage and the lower is grounded. The high voltage is an AC signal with a DC offset to maintain an oscillating and downward electric field between the two electrodes. This oscillating electric field is employed to initiate cone formation by destabilizing the droplet surface, while preventing the charge accumulation typically observed in conventional EHD, which would otherwise hinder MN formation. While being stretched in the oscillating field, droplet is gradually solidified and eventually transformed into a rigid MN after the solvent have been completely evaporated. In PIDES, the pulsed voltage signal consists of several adjustable parameters, that is, high voltage threshold (*Φ_h_*), low voltage threshold (*Φ_l_*), frequency (*f)* and duty cycle (Figure 1b). These parameters govern the deformation of the droplet into the Taylor cone as well as in preserving the MNs while the solvents are being diffused by evaporation.

## 3 Results and Discussion

**Figure 2:**
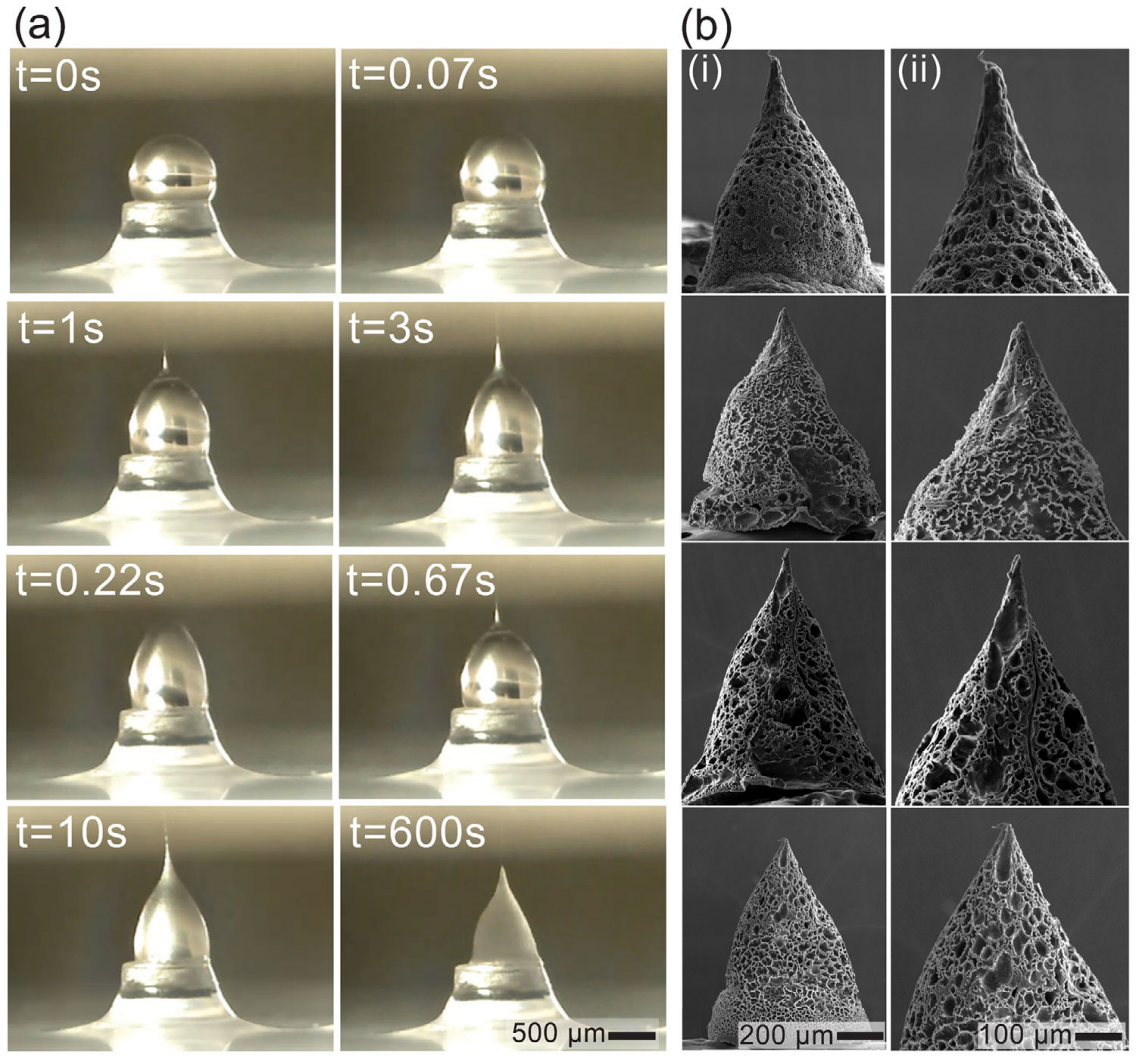
a) Temporal formation of a MN by PIDES; b) Scanning electron microscopy (SEM) images of 4 MNs: i) overall shape and ii) tip close‐up capture.

**Figure** **2**a presents the transient development of the MN from initial droplet state to the final solidified state in our PIDES approach (see Movie S1, Supporting Information). The instance t = 0s is captured before voltage is enabled, while t = 0.07s to t = 10s shows different stages of MN development and solidification. The MN has fully solidified and attained sufficient rigidity at t = 600s. At t = 0s, the polymeric droplet initially formed a spherical shape on the pedestal. After applied, the pulsed voltage swiftly destabilized, deformed and drew the droplet toward the upper electrode (t = 0.07s and 0.22s). The continuous voltage created the jet out of the meniscus (t = 0.67s) and formed the Taylor cone. From t = 1s to 10s, the conical shape was slightly elongated due to the voltage pulses and its transparency is visibly reduced due to the decrease in solvent fraction. As the solvent continued to evaporate, the cone shrank and solidified until it reached rigid stage (t = 600s). Statistical analysis on the geometric measurements of 20 MN samples by SEM captures shows an average tip diameter of 25 µm varying within a range of 11 – 46 µm which falls properly in the required tip diameter range for penetrating human tissues (10 – 50 µm).^[^ ^18^ ^]^ Other measurements include the needle's height and base diameter which were around 960 ± 124 µm and 735 ± 96 µm (standard deviation), respectively, resulting in an aspect ratio of 1.32. Notably, the fabricated MNs’ height is sufficient to penetrate the dermis layer and well under the pain‐causing threshold (1500 µm).^[^ ^16^ ^]^ The SEM images of 4 representative MNs are shown in Figure 2b‐i, and the close‐up views of their tip are included in Figure 2b‐ii). Furthermore, SEM images reveal randomly distributed macroporous structures throughout the MNs’ surface. This can happen when a polymer is dissolved in two solvents. The varying evaporation rates of the solvent cause phase separation, leading to the formation of polymer with high porosity.^[^ ^35^, ^36^ ^]^ Nevertheless, the porosity of the carrier benefits transdermal drug delivery by storing solid and liquid drug formulations, enhancing drug protection, release control, and solubility due to its large surface area.^[^ ^1^, ^37^ ^]^

**Figure 3:**
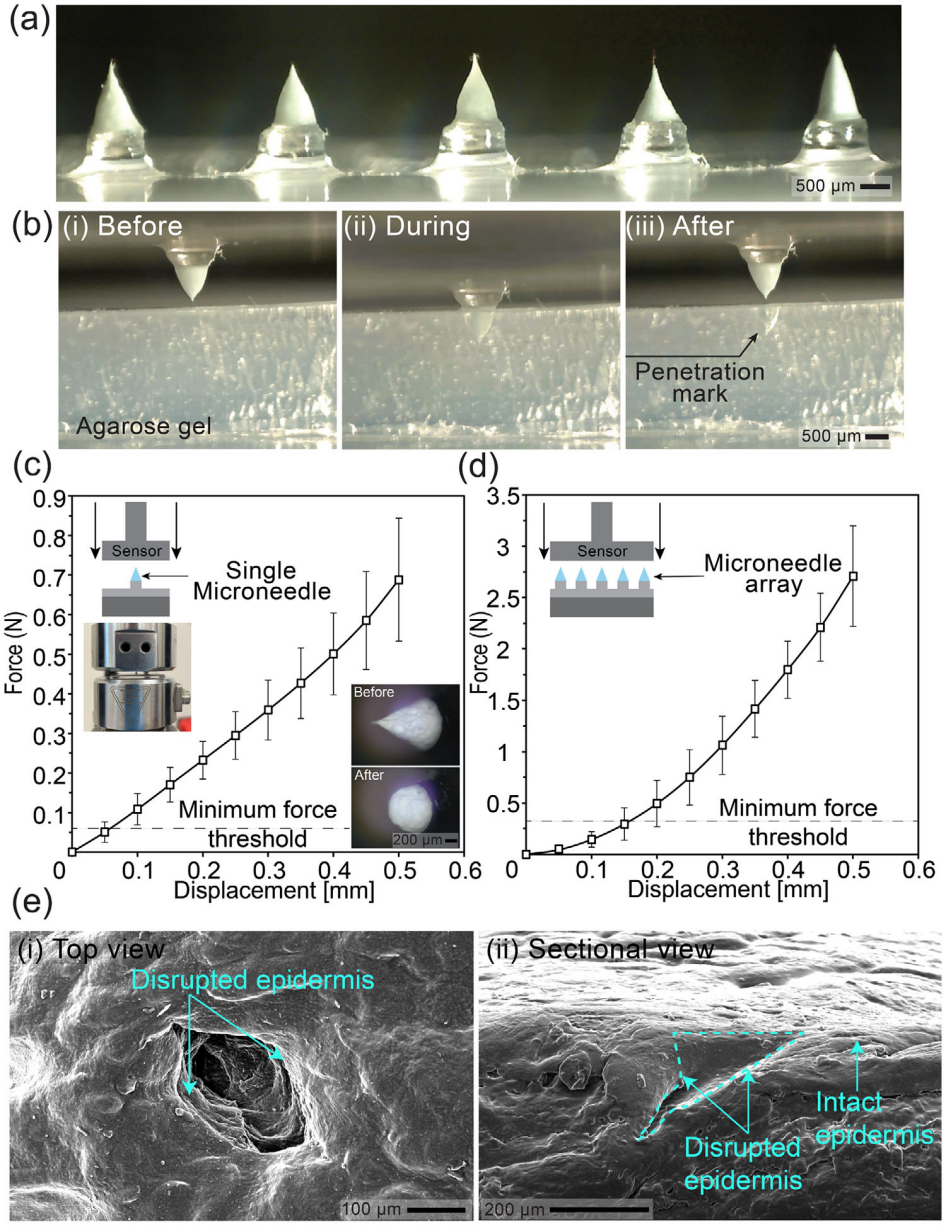
a) An array of MNs sequentially produced by PIDES; b) Different states of MN and agarose gel in the penetration test; c,d) Displacement variation with applied compressive force for: (c) Single fabricated MN; and (d) Array of 5 MNs. Data is mean±standard deviation ((c) n = 20 and (d) n = 4). Insets show c) illustrative setup (top left) and the shape of the MN sample before and after the mechanical test (bottom right); and d) illustrative setup; e) SEM captures of pig cadaver skin samples after ex vivo penetration test: i) top view and ii) sectional view.

To demonstrate reproducibility and scalability, we sequentially created an array of multiple MNs on the same substrate, as shown in **Figure** **3**a. All MNs in the array were formed with visibly sharp tips, with average height and aspect ratio of 1 mm and 1.3, respectively. This result indicates that the precedingly formed MNs does not influence the stretching of the succeeding MNs, inferring the ability of PIDES in creating more desired 2‐D arrays. However, geometric inconsistency, which is mainly associated with the variation of initial droplet volume in the manual dispensing step, can still be observed. Future development of an automatic dispensing and fabrication system may help overcome this shortcoming and enhance the applicability of our method.

Figure 3b shows different stages of the penetration test to demonstrate the stiffness of a fabricated MN, completely plunged through the agarose bed without any structural damage (Figure 3b‐ii). The MN remained relatively unscathed yet imprinted a clear penetration mark in the agarose medium after being retracted (Figure 3b‐iii) (see Movie S2, Supporting Information). Figure 3c shows the variation of displacement with applied force for individual MNs fabricated by our method. A single MN can sustain the required penetration force threshold of 0.058N^[^ ^38^ ^]^ with insignificant deformation. Specifically, typical PIDES MNs experienced an average of 6.5% height reduction under this minimum penetration force, thus demonstrating their practical mechanical strengths. In addition, the force‐displacement graph for an array of 5 MNs in Figure 3d shows an averaged displacement of 16% at the 0.29N threshold (five times of the minimum force threshold). Moreover, Figure 3e presents top and sectional views of the indentations created by MN penetration into the pig cadaver skin. The top‐down view clearly shows the disruption of the outermost epidermis layer which exposes the inner dermis layer of the skin sample, whereas the sectional view further reveals a triangular cavity resembling the shape of the MN. These results show that the MNs can sustain considerable compressive force and effectively penetrating real skin samples, demonstrating their functionality as well as the potential of the PIDES method in transdermal drug delivery.

The above results have shown that PIDES technique can create suitable MNs in a one‐step in situ fabrication process using a simple parallel‐plate setup. This feature could overcome the disadvantages of the common mold‐based methods which are multi‐step process, from master molds casting, material filling and MN detaching or transferring.^[^ ^6^, ^15^, ^17^, ^22^, ^23^, ^24^, ^25^, ^26^ ^]^ Moreover, although current moldless methods, such as liquid drawing, wet etching, 3D printing and pyroelectric electrodrawing, involve fewer steps, they require microfabrication technologies or electronically controlled dispensing and pulling systems, or the utilization of pyroelectric crystal to create MNs.^[^ ^4^, ^7^, ^18^, ^27^, ^28^, ^29^, ^30^, ^31^, ^32^, ^33^ ^]^

**Figure 4:**
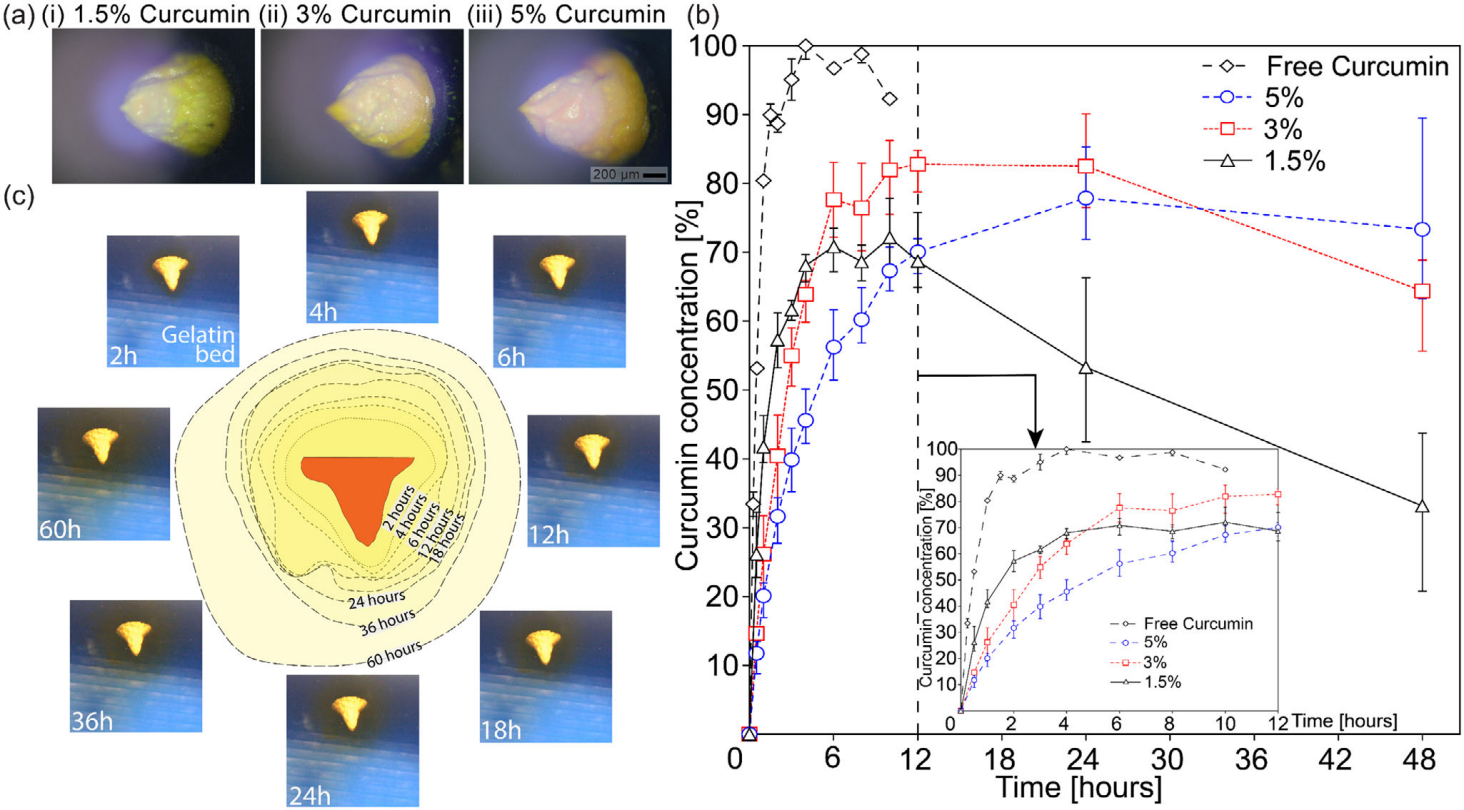
a) Inclined capture of MNs with different curcumin concentrations showing different shades of yellow indicating different curcumin densities; b) Curcumin release profile of curcumin‐loaded MNs at 1.5%, 3% and 5% w/w concentrations. Data is the means with error bars indicating the range of measured values (triplications, with each repetition involves n = 50 MN samples). Inset shows release profile of these samples in the first 12 h; c) Time‐lapsed captures of curcumin release in gelatine gel bed.

**Figure** **4**a shows the inclined capture of MN produced from different curcumin concentrations. These results have demonstrated that our method is able to produce MNs carrying different concentrations of curcumin while preserving the tip's sharpness, consolidating the potential of PIDES for transdermal drug delivery. Figure 4b shows the release profile of free curcumin and curcumin loaded in the MNs with different w/w concentrations (1.5%, 3% and 5%). The release kinetics of curcumin encapsulated in the MNs in all concentrations exhibited a linear drug release profile in the first 6 h and achieved maximum curcumin release of more than 70%. These release profiles demonstrate a controlled release of curcumin by the MNs, differentiating from the burst release of free curcumin (90% in 1.5 h, shown in Figure 4b inset). In addition, the results yield faster release rates as curcumin concentration is reduced and a more sustained release as concentration is increased. Particularly, the 1.5%, 3% and 5% w/w cases achieved their maximum release concentrations after 10, 12, and 24 h, respectively. After such time points, the curcumin concentration of the 1.5% w/w case decreased quickly, while the 3% and 5% w/w cases experienced more gradual decreases. The reductions in curcumin concentration in the release media can be due to curcumin's degradation at 37 °C.^[^ ^39^ ^]^ According to these results, the 3% w/w concentration can be considered most efficient because of its highest maximum release, while the 5% w/w concentration is more applicable in cases where a prolonged and stable release kinetics is required. Moreover, the diffusive release of curcumin (3% w/w) in gelatin gel is presented in Figure 4c. The results demonstrated a continuous release over 60 h (see Movie S3, Supporting Information), indicating that our drug‐loaded microneedles (MNs) are highly compatible with human tissue.

**Figure 5:**
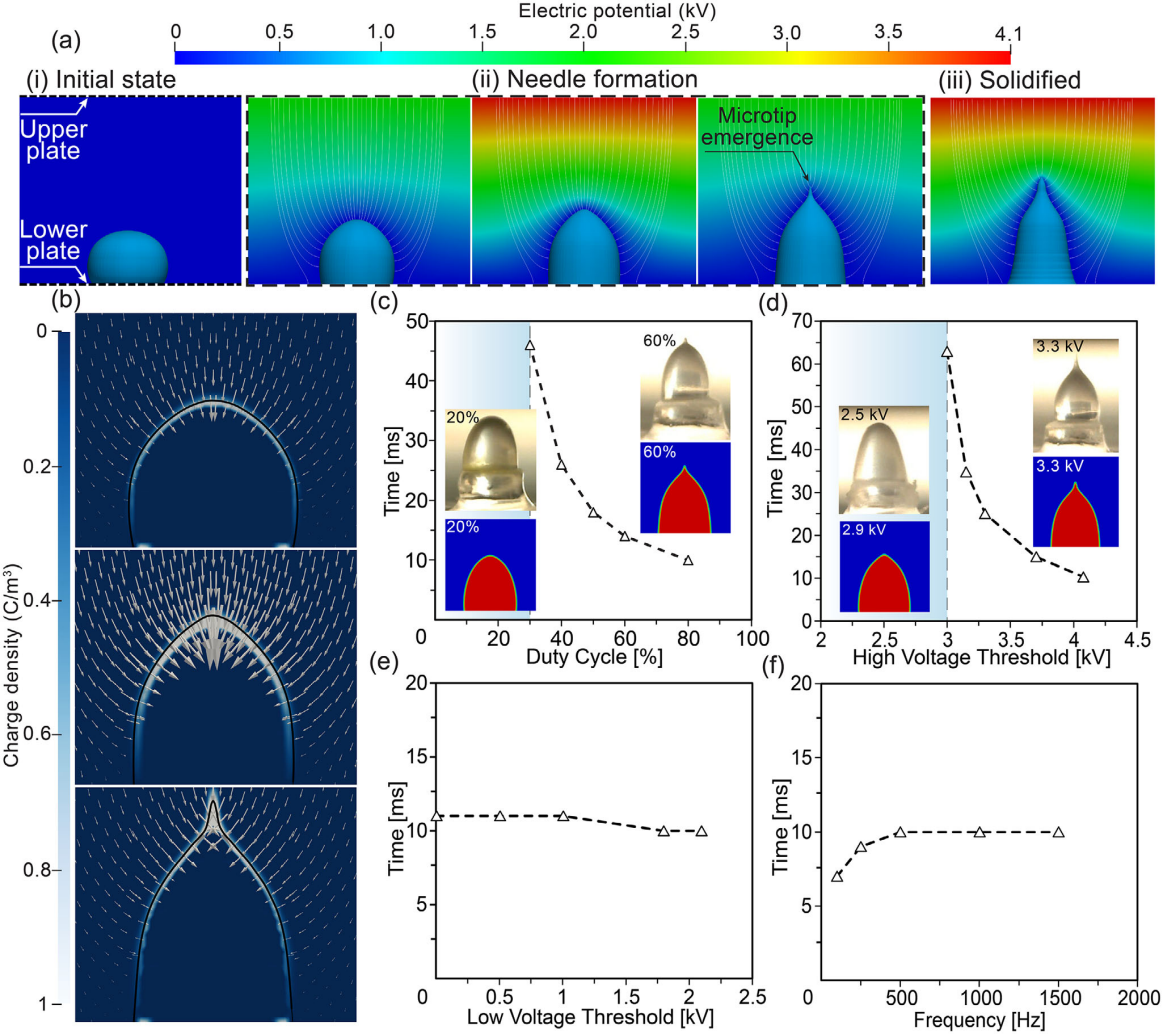
a) Numerical reproduction of the temporal MN formation at different state of voltage signal, a‐i) Initial state shows static droplet without voltage signal between the upper and lower plate boundaries; a‐ii) Needle formation shows different development of a microtip; a‐iii) Solidified MN state at 0.4 µL; background contour indicates electric potential field, streamline represent electric field. b) The behavior of polarized charge accumulation (background contour) and electric field (vector) at different stages of MN formation, with black line represents the droplet interface. c–f) Simulation results of the effect of different parameters on the formation of the microtip (c) Duty cycle (fixed parameters: Φ_h_ = 4.1 kV, Φ_l_ = 2.1 kV, f = 500 Hz); d) High voltage threshold (fixed parameters: Φ_l_ = 2.1 kV, f = 500 Hz, duty cycle = 80%); e) Low voltage threshold (fixed parameters: Φ_h_ = 4.1 kV, f = 500 Hz, duty cycle = 80%); f) Frequency cycle (fixed parameters: Φ_h_ = 4.1 kV, Φ_l_ = 2.1 kV, duty cycle = 80%).

**Figure** **5**a shows the simulation of the development of a liquid droplet into a microneedle shape. In this case, we used a two‐stage simulation with two different viscosities. We commenced with an initial viscosity of 0.268 Pa.s to model the formation of the microtip from a droplet. Once the microtip is formed, we increased the viscosity to 72 Pa.s (equivalent to 75% PCL w/w, see Supporting Information S2) to simulate the continued shrinkage due to solvent evaporation. As seen in Figure 5a‐ii, the liquid droplet was quickly deformed and heightened by the influence of the voltage signal, eventually giving rise to a conical shape with a microtip. As viscosity increased, the shape solidified and relatively unaffected the voltage pulses, yet still lost mass due to the effects of evaporation. Figure 5a‐iii shows a solidified MN shape at 0.4 µL, after 20% of the original liquid mass has been transferred to vapor. Similar to experimental observations, the continuous evaporation of solvent mostly resulted in width reduction, streamlining the MN for better shape and penetrability. Additionally, Figure 5b shows the negative surficial charge accumulation as a result of the dielectric polarization effect and the electric field direction in the vicinity of the droplet surface. The negative charge accompanied by inward electric field result in outward Coulombic force, causing the transformation of the liquid droplet into the MN shape.

The emergence of the microtip depends on the governing voltage parameters including duty cycle, high voltage threshold (*Φ_h_*), low voltage threshold (*Φ_l_*), and frequency (*f*). The effects of these parameters on the microtip formation were numerically investigated. Figure 5c–f shows the correlation between microtip emergence time and voltage parameters. Duty cycle and *Φ_h_* significantly influence microtip formation (Figure 5c,d), with minimum duty cycle and minimum high voltage threshold (*Φ_h_)* should be greater than 20% and 3 kV, respectively for tip formation. Increasing duty cycle and *Φ_h_* leads to an exponential reduction in tip emergence time. When the duty cycle is too low, the voltage is insufficiently long to overcome the liquid's shape recovery. At higher duty cycles, the longer high‐voltage overwhelms the recovery of liquid meniscus and is able to form a microtip. For *Φ_h_*, if the threshold is below the onset value for Taylor cone formation, a microtip cannot be formed. Although these simulation results are useful for estimating the conditions required for microtip formation, important physical principles such as the temporal solidification of the liquid as well as the polymeric inhomogeneity in the evaporation process are currently neglected. This could be addressed in future work to improve the model's accuracy.

## 4 Conclusion 

In conclusion, a novel technique is proposed to fabricate polymeric MNs for transdermal drug delivery applications. Experimental results showed that the PIDES method successfully produced MNs with consistent shapes and sizes suitable for penetrating the dermis layer for drug release. The sequential MNs fabrication indicated the repeatability of our techniques, whereas agarose gel penetration and mechanical strength tests confirmed the stiffness of the solidified polymeric MNs. Results from curcumin loading and in vitro release kinetics showed a reasonable controlled release of drug in the MNs as well as a practical compatibility with the human transdermal conditions of the drug‐loaded MNs. Numerical investigations provided a deeper understanding of the phenomenon as well as predictions on parametrical relationships between the pulsed voltage signal and the ability to form a microtip. In future works, optimization investigation and the development of an on‐demand MN array fabrication method will be carried out to consolidate the applicability of our novel technique.

## 5 Numerical Model

We develop an OpenFOAM code based on the Taylor‐Melcher leaky dielectric model to simulate PIDES process.^[^ ^46^, ^47^ ^]^ Our numerical solver is developed in the open‐source OpenFOAM package. The Taylor‐Melcher leaky dielectric model is further implemented into the source code of this solver. This model incorporates the Navier‐Stokes equations, the Gauss's Law and the charge conservation equation to simulate a liquid medium placed in an electric field. The fundamental governing equations are the continuity and the momentum equation for the fluid‐dynamic regime is,

(1) $$\frac{\partial \rho }{\partial t}+\nabla \cdot \;\left(\rho u\right)=0$$

(2) $$\rho \left[\frac{\partial u}{\partial t}+\left(u\cdot \nabla \right)u\right]=-\nabla p+\mu \nabla^{2}u+f_{\sigma }+f_{e}+\rho g$$

whilst electrostatic regime is governed by the Poisson's equation and the charge conservation equation,

(3) $$\begin{array}{c} \nabla^{2}\;\left(\phi \right)=\frac{-\rho_{e}}{\varepsilon } \end{array}$$

(4) $$\frac{\partial \rho_{e}}{\partial t}+\nabla \cdot \left(\rho_{e}u\right)+\nabla \cdot \;\left(\kappa_{e}E\right)=0$$

Finally, the electrostatic force can be considered as the summation of Coulombic force and polarization force with electric field relation with potential ***E *** =   − ∇ϕ,

(5) $$\begin{array}{c} \;f_{e}=\rho_{e}\;E-\frac{1}{2}{\left|E\right|}^{2}\nabla \varepsilon \end{array}$$

Additionally, we employed a diffusion evaporation model provided by OpenFOAM package (diffusionGasEvaporation) to consider the volumetric variation of the MN, which includes the energy equation for incompressible flow and the d^2^ Law^[^ ^48^ ^]^ for evaporation mass transfer,

(6) $$\frac{\partial }{\partial t}\left(\rho c_{p}T\right)+\nabla \cdot \;\left(\rho c_{p}uT\right)=\nabla \cdot \left(\lambda \nabla T\right)+S$$

(7) $$\begin{array}{c} \;\hat{m_{i}}=-C\rho_{g}D_{v,i\;}\frac{\frac{dY_{v,i}\;}{dn}}{1-\sum_{1}^{N_{v}}Y_{g,j}} \end{array}$$

With *ρ* is fluid density, *t* is time, ***u*** **is fluid velocity, *p* is pressure, *µ* is fluid viscosity, and ***g*** is gravitational acceleration, ***f*** *_σ_* and ***f*** *_e_* are surface tension force and electrostatic force, respectively, *ɛ* is permittivity of the fluids, ***E*** is electric field, ϕ is electric potential and *ρ_e_* is volumetric charge density, *κ_e_* is electric conductivity. Additionally, *c_p_* is specific heat capacity of the fluids, *T* is temperature field, *λ* is thermal conductivity, ***S*** indicates the source term, typically neglected in cases without internal heat source. In the evaporation model (Equation 7), $\hat{m_{i}}$ presents the mass flux transferred from liquid state to the gas state, *C* is the model coefficient, *ρ_g_* is gas phase density, *D_v,i_* is diffusion coefficient, d*Y_g,j_/dn* is interfacial normal derivative of evaporated component, *Y_g,j_* is gas mass fraction at the surface. Please see Supporting Information S1 for additional information on simulation domain and boundary conditions.

In our simulations, we assume that the rigidity of the liquid is mainly dictated by its viscosity whose relationship with the polymer concentration can be represented by the Huggins equation.^[^ ^49^ ^]^ This relation can be determined by using the Oswald viscometer to measure the viscosities of polymeric solution (0.2683 Pa.s for PCL 15%) (see Supporting Information S2). This value was used in simulations while other liquid properties were referenced from Acetone's properties at 25 °C (surface tension *σ* = 24.5 mNm^−1^, density ρ = 0.784 g mL^−1^, electric conductivity *κ_e_* = 2 × 10^−5^ Sm^−1^, relative permittivity *ɛ_r_* = 16.2, specific heat capacity *c_p_* = 2.14 kJkg^−1^K^−1^, thermal conductivity *λ* = 0.18 Wm^−1^K^−1^). The initial liquid droplet was set to 0.5 µL in volume and was modeled to form a 120° contact angle with the lower plate boundary, reflecting the hydrophobicity of the experimental substrate.

## 6 Experimental Section

### MN Fabrication

**Figure 6:**
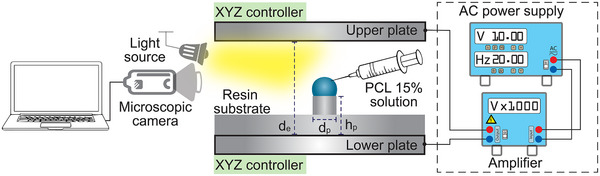
The experiment installation of MN fabrication by PIDES.

To prove the feasibility of our concept, we designed an experimental apparatus as shown in **Figure** **6**. Polycaprolactone (PCL – Sigma‐Aldrich, average molecular weight of 80 000 g mol^−1^), was dissolved in a mixture of Acetone (Sigma‐Aldrich) and Dimethylformamide (DMF – Sigma‐Aldrich) in a 7:3 w/w ratio to create a PCL 15% solution. A sub‐microliter (≈0.5 µL) polymeric droplet of the solution was dispensed by a plastic syringe (Terumo) extended by a 30‐gauge nozzle (Musashi Engineering) between two parallel NiCu electrodes attached to an electrically controlled XYZ stage (0.1 mm step resolution) to adjust and control the interelectrode distance (d_e_). The voltage signal was generated by a signal generator (RIGOL) and an amplifier (Advanced Energy). The formation and solidification process of the MN were recorded by a digital microscopic camera (Dino‐lite EDGE) connected to a computer for real‐time video capturing and data processing.

The experiment was conducted at room temperature (25 °C), with 40–60% humidity and atmospheric pressure. The conditions *Φ_h_* = 4.1 kV, *Φ_l_* = 2.1 kV, *f* = 500 Hz, duty cycle = 80%, and an inter‐electrode distance of 5.5 mm were maintained throughout all experiments. These parameters were found to yield a desirable MN shape and ensure a stable, repeatable MN formation process.

### Scanning Electron Microscopy

SEM images was collected with a Phenom XL G2 Desktop SEM 15 kV beam intensity and secondary electron detector (SED) mode. Samples were attached to SEM stubs by carbon tape and sputtering coated with gold for 3 min (NeoCoater).

### Mechanical Strength

A thin sheet of agarose gel 4%, casted from agarose powder (BioRad) in 1× TAE buffer (Biorad), was used as testing material due to its controllable mechanical properties and transparency for monitoring the depth of penetration.^[^ ^7^ ^]^ The MN penetrated at a vertical speed of 5 mm ^−1^s and a depth of 1 mm. The mechanical strength of the MNs was further investigated by a compressive test using a desktop displacement force station (Instron 3367) with an axial translating rate of 0.5 mm min^−1^.

### Ex Vivo Pig Cadaver Skin Penetration

Consumer‐grade pig cadaver skin samples were purchased and stored in −20 °C freezer to retain freshness and thawed to normal state before the experiment. To mimic the texture and toughness of live tissues, the skin samples was submerged into a 37 °C water bath for 10 min before the penetration test. The MN and skin samples were attached and sandwiched between two parallel glass slides (Supporting Information S5) pressed together with normal thumb force for 60 s. The glass slides were then detached, and a sectional view was attained by slicing the skin sample by a scalpel. The samples were then dehydrated by submerging for 4 h in Ethanol with increasing concentrations (70%, 90%, 95%, 100%) and left dried overnight in a fume hood. The dried skin samples were then investigated by SEM as previously presented.

### Curcumin Loading and In Vitro Drug Release Kinetics

Curcumin, a yellow hydrophobic pigment derived from turmeric, is widely used in biomedical applications.^[^ ^40^ ^]^ Known for its anticancer and anti‐inflammatory properties, curcumin is ideal for transdermal delivery in wound regeneration,^[^ ^41^ ^]^ cancer prevention,^[^ ^42^ ^]^ and treatment of various health issues.^[^ ^43^, ^44^ ^]^ Curcumin (Sigma Aldrich, from Curcuma longa) was added into the PCL 15% solution at different concentrations, that is, 1.5%, 3% and 5% w/w to demonstrate the drug‐loading capability of the polymeric MNs produced by PIDES.

Fifty curcumin‐loaded MNs of each concentration (1.5%, 3% and 5% w/w) and free curcumin were dissolved separately in 100 mL of the release media, phosphate‐buffered saline (PBS, Sigma‐Aldrich) – Tween 0.5% solution (pH 7.4, Polysorbate 80, Sigma‐Aldrich), at 37 °C and stirred 200 rpm in a magnetic stirrer.^[^ ^40^ ^]^ In total, 900 MN samples with varied concentrations were fabricated and used in the drug release experiments. At predetermined time intervals of 30 mins, 1, 2, 3, 4, 6, 8, 10, 12, 24, and 48 h, 2 mL of released sample from curcumin‐loaded MNs was withdrawn and replaced with fresh release media. The sampling intervals for free curcumin were 15 mins, 30 mins, 1, 1.5, 2, 3, 4, 6, 8, and 10 h. The concentration of the released curcumin was determined using UV‐1800 (Shimadzu Scientific Instruments) and a standard curve of known curcumin concentrations in the release media by measuring absorbance at a wavelength 425 nm.^[^ ^40^ ^]^ Free curcumin and curcumin‐loaded MNs tests were triplicated to ensure the reliability of the measurements. The total curcumin concentration was determined by dissolving fifty curcumin‐loaded MNs in 100 mL ethanol (≥99.5%, ACS reagent, Sigma Aldrich). The release speed in the solution was accelerated using an ultrasonic bath. After 30 min, three 2 mL samples from the release solution were drawn and measured by the UV‐1800 spectrophotometer. This procedure was also triplicated to ensure the best reliability of the total curcumin concentration value. The measured values were later averaged to obtain the final results. All spectrophotometric measurements were performed employing quartz cuvettes (Hellma).

Gelatin gel, known to simulate the density and viscosity of human tissue,^[^ ^45^ ^]^ was created by mixing gelatin powder (from porcine skin, gel strength 300, type A, Sigma‐Aldrich) 10% w/w with PBS‐Tween 3% solution to mimic the human physiological conditions. The mixture was vigorously stirred at 2000 rpm and heated to 50 °C for quick dissolution of gelatin. After the solution became homogeneous and gelatin had completely dissolved, the solution was injected into the gelatin bed (see Supporting Information S4) and left cooled off to become a gel. The curcumin‐loaded MN was then inserted into the bed which was sealed with parafilm to prevent the gel from being dried out and solidified. Timelapse video capture was performed using a microscopic camera (Dino‐lite EDGE).

### Statistical Analysis

The sample size (n) and data presentation were included in the corresponding figure legends and captions. All data was processed using Excel (Microsoft Corporation).

## Conflict of interest

The authors declare no conflict of interest.

## Supporting information



Supporting Information

Supplemental Movie 1

Supplemental Movie 2

Supplemental Movie 3

## Data Availability

The data that support the findings of this study are available from the corresponding author upon reasonable request.
