# Supplementary material for: Fabrication of Microneedles by Pulsating In Situ Dried Electrostretching for Transdermal Drug Delivery
Source: Small Methods. 2025 May 7;9(10):2500183. doi: 10.1002/smtd.202500183 (PMC12536382; doi:10.1002/smtd.202500183)
Supplement: Supplementary file 1 — Supporting Information [file SMTD-9-2500183-s003.docx]

Supporting Information

Fabrication of microneedles by pulsating in-situ dried electrostretching for transdermal drug delivery

Ngoc Luan Mai^*^, Yuen Yong^**^, Thi Van Anh Hoang, Trung Hieu Vu, Hoai-Duc Vu, Van Canh Doan, Donglin Cai, Thien Xuan Dinh, Dzung Viet Dao, Van Thanh Dau^***^

**S1. Simulation models**


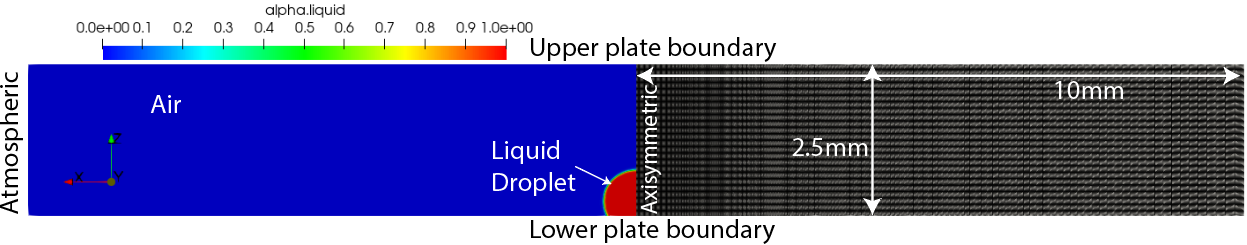


Figure S1. The computational domain with boundaries and mesh model of MN simulations.


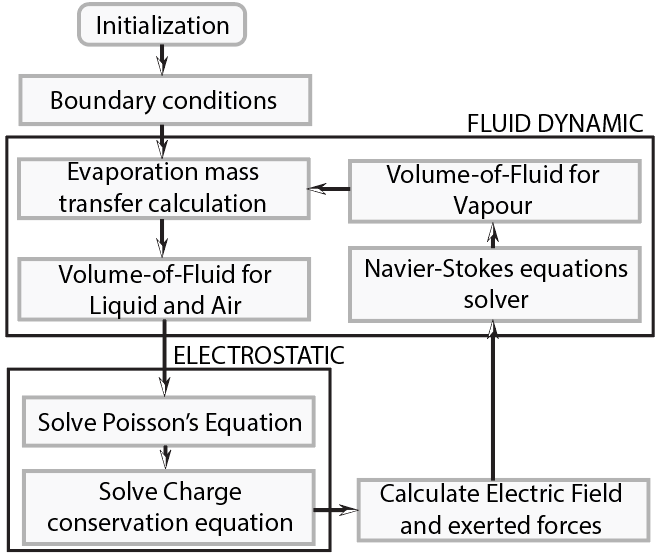


Figure S2. Flowchart of the numerical solver.

The mesh model was constructed using blockMesh which is another built-in module of OpenFOAM. In this case, the total number of cells of 26,500 was obtained as minimum cell size is kept at $20 \mu m$. The mesh model and computational domain is shown in Fig. S1. Additionally, Tab. ST1 provides the boundary conditions for each boundary in our simulations. A detail description of the solution process is provided in Fig. S2. The electrostatic and fluid dynamics equations are solved sequentially, with the evaporation phase change calculations occurring at each timestep.

Table ST1. Boundary conditions for the computational domain in physical parameters.

| **Boundary** | **Fluidic conditions** | **Electrostatic conditions** | **Thermophysical conditions** |
| --- | --- | --- | --- |
| Upper plate | $u_{x}=u_{z}=0$ (no slip); $\theta=\theta^{\circ}$ | $\phi=\phi_{pulse}$ | $\nabla T=0$ |
| Lower plate | $u_{x}=u_{z}=0$ (no slip); $\theta=\theta^{\circ}$ | $\phi=0$ | $\nabla T=0$ |
| Atmospheric | $p=p_{0}$ | $\nabla\phi=0$ | $T=T_{0}$ |
| Axisymmetry | $\frac{{du}_{z}}{dx}=0$ | $\frac{d\phi}{dx}=0$ | $\frac{dT}{dx}=0$ |

**S2. Viscosity measurement and estimation**

Since the addition of polymer concentration into solvents may significantly increase the viscosity of the mixture, we perform empirical measurements to determine the viscosity of the employed PCL 15% polymeric solution. Using an Ostwald viscometer, we obtained the time required by the liquid to flow between two-mark points which was later used to calculate the kinematic viscosity of the liquid. By further measuring the density of the solution, we were able to calculate the dynamic viscosity of liquid. These calculations were based on the following formulas,

$$\begin{aligned} \nu=\kappa\Delta t, \#\left( S1 \right) \end{aligned}$$

$$\begin{aligned} \mu=\rho\nu, \#\left( S2 \right) \end{aligned}$$

where $\nu$ is kinematic viscosity, $\kappa$ is the viscosity coefficient provided by the device’s manufacturer, $\Delta t$ is the measure time, $\mu$ is the dynamic viscosity, and $\rho$is density.

To ensure the objectivity of the method, we measured the required flow time and density of the solution three times consecutively. Obtained values were later averaged out to determine the final value.

Moreover, as we assumed that the solidification of the Taylor-cone as well as the polymeric solution is dictated by the gradual increase in viscosity, we performed additional measurements on the viscosity of the pure-solvent mixture (Acetone and DMF 7:3) which followed the same procedure with the PCL 15% viscosity measurement. Tab. ST2 shows the acquired and average density and viscosity values.

Table ST2. Results for viscosity measurement for pure-solvent and PCL 15% solution.


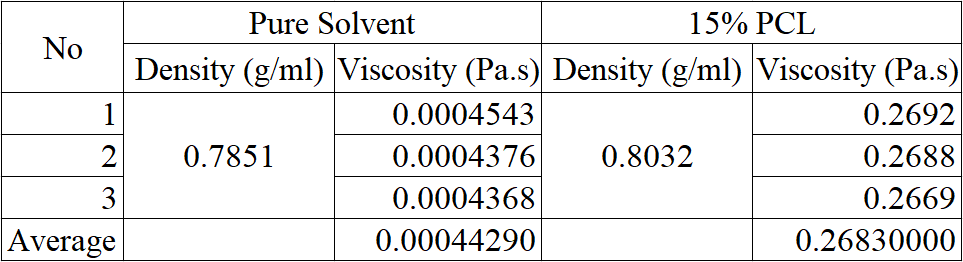


We carried on using the Huggins equation [1] to determine the correlation between the polymer concentration and the viscosity for the solution,

$$\begin{aligned} \frac{\eta_{s}}{c}=\left[ \eta\right]+k_{H}\left[ \eta\right]^{2}c,\#\left( S3 \right) \end{aligned}$$

in which $\eta_{s}=$ $\eta_{r}-1=\frac{\eta}{\eta_{0}}-1$is the specific viscosity of a solution at a given concentration of a polymer in solution, $\left[ \eta\right]$ is the intrinsic viscosity of the solution, $k_{H}$ is the Huggins coefficient, and c is the concentration of the polymer in solution. We rearrange Eq. S3 as follows,

$$\begin{aligned} \frac{\eta}{\eta_{0}}=\left[ \eta\right]c+k_{H}\left[ \eta\right]^{2}c^{2}+1,\#\left( S4 \right) \end{aligned}$$

Taking the pure-solvent viscosity as $\eta_{0}$ and the PCL 15% viscosity as $\eta$ and $c=0.14576 g/ml$ (equivalent to PCL 15% w/w) and $k_{H}=0.3$ for strong solvents, we can calculate the intrinsic viscosity $\left[ \eta\right]$. This calculated value was later used in Eq. S4 whilst keeping $c$ as variable and $\eta$ as function, we can determine the viscosity-polymer concentration plot shown in Fig. S3.


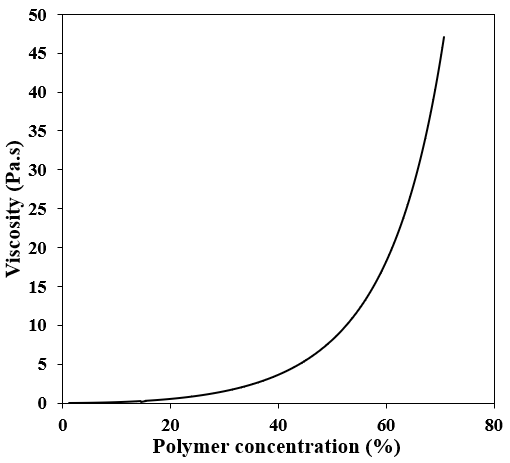


Figure S3. Viscosity-Polymer concentration correlation graph for PCL 15% in Acetone:DMF 7:3 w/w solution.

**S3. Confocal capture of curcumin released in gelatin gel**

**
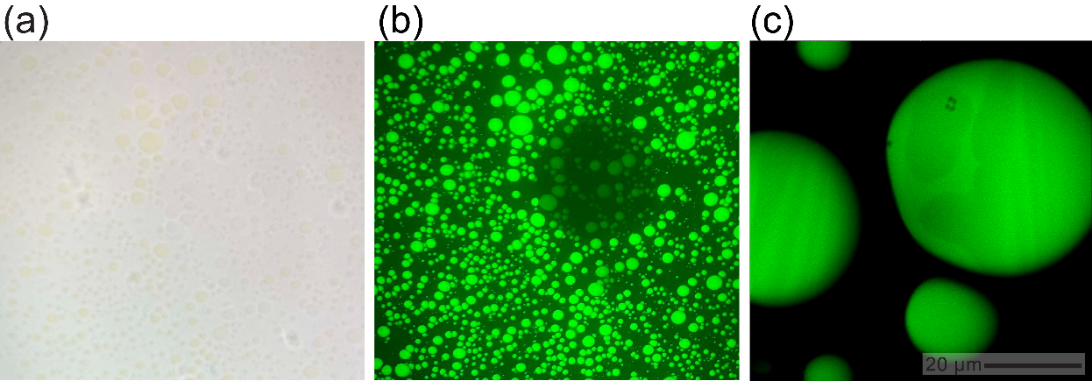
**

Figure S4. Gelatin gel containing released curcumin captured via (a) diascopic illumination; (b) epi-illumination using fluorescein isothiocyanate mirror (FITC); (c) LSM captured of curcumin trapped in gelatin using Lucifer Yellow dye’s parameters [2].

Curcumin released from the MN into the gelatin bed is visualized by laser scanning microscopy (LSM), shown in Fig. S4. The design of the gelatin gel bed is presented in section S. After curcumin has been released to gelatin gel, we slice a small piece of the curcumin-rich gelatin gel and place it between a microscope glass slide and a coverslip. Then, the object was slightly heated up so the gel can be melted into a very thin sample that can be viewed by LSM microscope (Olympus FV3000). Fig. S4(a) shows the melted gelatin gel viewed with diascopic illumination from which yellowish bubbles can be observed. The bubbles were caused by the melted gelatin while the yellow regions indicate high curcumin content. Additionally, curcumin presence in the sample was clearly illuminated using epi-illumination using fluorescein isothiocyanate mirror (FITC-$\lambda_{\mathrm{exc}}=495 nm,\lambda_{\mathrm{emis}}=519 nm$ [3]), showing the same curcumin-rich bubble structures (Fig. S4(b)). Finally, close-up view of curcumin-rich bubbles is shown using LSM and Lucifer Yellow dye’s excitation and emission wavelengths (Fig. S4(c)) ${(\lambda}_{\mathrm{exc}}=428 nm,\lambda_{\mathrm{emis}}=530 nm$ [2]) which are similar to those of Curcumin [4].

**S4. Gelatin gel bed preparation**


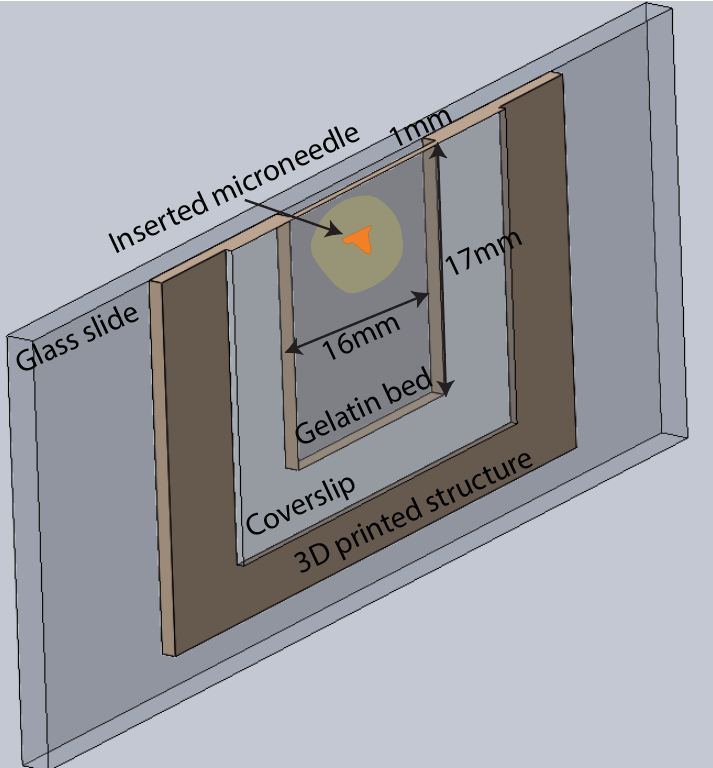


Figure S5. The design of the gelatine bed used for curcumin-load MN release test.

The design of the gelatin gel bed is shown in Fig. S5. The gelatin bed was sandwiched between a microscope glass slide and a coverslip and enclosed by a 3D-printed structure with pre-determined sizes to leave sufficient space for curcumin release.

**S5. Ex-vivo pig cadaver skin penetration experiment**

Fig. S6 shows the arrangement, design and captures of the ex-vivo pig cadaver skin penetration test as presented in more details in the main manuscript.

*
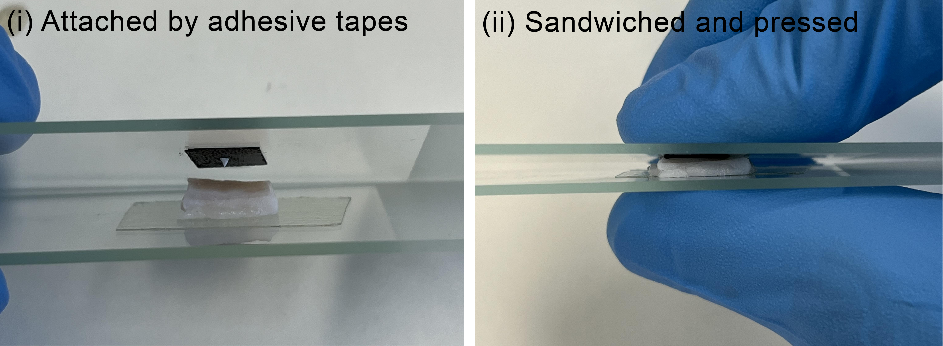
*

Figure S6. Arrangement, design and captures of the ex-vivo pig cadaver skin penetration test: (i) MN and pig skin sample are attached to two parallel glass slides and (b) Two glass slides are sandwiched and pressed by normal thump force.

**REFERENCE**

[1] H. M. Kwaambwa, J. W. Goodwin, R. W. Hughes, and P. A. Reynolds, "Viscosity, molecular weight and concentration relationships at 298K of low molecular weight cis-polyisoprene in a good solvent," *Colloids and Surfaces A: Physicochemical and Engineering Aspects,* vol. 294, no. 1, pp. 14-19, 2007/02/15/ 2007, doi: <https://doi.org/10.1016/j.colsurfa.2006.07.041>.

[2] T. Clark Brelje, M. W. Wessendorf, and R. L. Sorenson, "Chapter 5 - Multicolor Laser Scanning Confocal Immunofluorescence Microscopy: Practical Application and Limitations," in *Methods in Cell Biology*, vol. 70, B. Matsumoto Ed.: Academic Press, 2002, pp. 165-249e.

[3] G. T. Hermanson, "Chapter 10 - Fluorescent Probes," in *Bioconjugate Techniques (Third Edition)*, G. T. Hermanson Ed. Boston: Academic Press, 2013, pp. 395-463.

[4] Z. Ali, M. Saleem, B. M. Atta, S. S. Khan, and G. Hammad, "Determination of curcuminoid content in turmeric using fluorescence spectroscopy," *Spectrochimica Acta Part A: Molecular and Biomolecular Spectroscopy,* vol. 213, pp. 192-198, 2019/04/15/ 2019, doi: <https://doi.org/10.1016/j.saa.2019.01.028>.
